# Sacral Alar-Iliac (SAI) screw diameter is not associated with pelvic fixation failure for neuromuscular scoliosis patients

**DOI:** 10.1007/s43390-025-01044-9

**Published:** 2025-01-30

**Authors:** Tyler A. Tetreault, Annika Y. Myers, Jaqueline Valenzuela-Moss, Tishya A. L. Wren, Michael J. Heffernan, Lindsay M. Andras

**Affiliations:** Jackie and Gene Autry Children’s Orthopedic Center, Children’s Hospital Los Angeles, 4650 Sunset Blvd, MS69, Los Angeles, CA 90027 USA

**Keywords:** Pediatric neuromuscular scoliosis, Pelvic fixation, Screw failure, Screw diameter

## Abstract

**Purpose:**

Determine if Sacral Alar-Iliac (SAI) screw diameter is associated with pelvic fixation failure in pediatric patients with neuromuscular scoliosis (NMS) treated with posterior spinal fusion (PSF).

**Methods:**

NMS patients from a single institution who underwent PSF with bilateral SAI screw fixation from 2010 to 2021 were retrospectively reviewed. Clinical parameters, SAI screw sizes, and radiographic outcomes were analyzed. Patients with greater or less than two SAI screws, > 21 years old, or with < 2 years of radiographic follow-up were excluded.

**Results:**

142 patients had 284 SAI screws placed. Mean(± SD) age was 13.6 ± 2.7 years. Preoperative curve magnitude averaged 84.3 ± 29.1°. Mean patient weight was 36.4 ± 14.1kg and BMI was 18 ± 5.1. Radiographic follow-up averaged 4.6 ± 2.0 years. Most screws (234/284,82.4%) were < 8.5 mm and 7.5 mm screws were most frequently used (158/248,55.6%). Mean screw diameter was 7.4 ± 0.7 mm. Patients with greater age, weight, and BMI trended towards larger screws. Three patients had five screw complications (1 screw fracture and 4 set screw dislodgments). One screw fracture (1/284,0.4%;7.5 mm diameter) and contralateral set screw dislodgement occurred in a patient at 14 months but was not revised. One patient who had bilateral set screws dislodge 3 months after PSF underwent revision. The remaining patient was asymptomatic and was observed. Screw diameter was not associated with risk of postoperative complications (*p* = 0.245).

**Conclusion:**

SAI screw fracture is rare after PSF in pediatric patients with NMS. Contrary to pelvic fixation in adults, smaller diameter SAI screws, which may be optimal in patients with smaller anatomy, were not associated with increased risk of screw failure.

**Level of evidence:**

III.

## Introduction

Neuromuscular scoliosis (NMS) is a spinal deformity that develops from a variety of conditions that affect brain, spinal cord, or muscle function. Cerebral palsy is the most common etiology, with 80% of patients who function at a Gross Motor Function Classification System (GMFCS) Level V developing NMS by 20 years old [1, 2]. Curves are typically progressive and can result in worsening seating balance and pulmonary and cardiac dysfunction [3]. Posterior spinal fusion (PSF) to the pelvis is often indicated to prevent further curve progression, improve seating balance, and improve patient and caregiver quality of life [4–6].

Pelvic fixation is a powerful method to correct pelvic obliquity and improve seating balance. Since first described in 2007, the sacral alar iliac (SAI) screw has gained popularity over the iliac screw (IS) technique due to a more favorable complication profile, particularly with screw prominence, wound breakdown, screw loosening, and need for revision surgery [7–11]. However, challenges with screw fracture persist. In the adult spinal deformity population, screw fracture rate on meta-analysis was 5.6% [12]. Similarly, in the pediatric population, screw fracture rates of 3.1–11.2% were noted in early case series, all of which occurred with SAI screws that were ≤ 8 mm in diameter [11, 13, 14]. Because of these findings and a positive mathematical relationship between screw diameter and lateral bending strength, in both adults and children it is recommended that screws with an outer diameter ≥ 9 mm be used during SAI fixation [15]. However, as pediatric neuromuscular patients often have smaller or atypical pelvic anatomy, we have frequently used smaller-diameter screws at our institution. In addition, with a lower body weight and lower physical demand than the adult population, the validity of the recommendation for ≥ 9 mm screws remains unknown. This study seeks to evaluate whether screw diameter is a risk factor for SAI screw failure in spinopelvic fixation of pediatric patients with neuromuscular scoliosis.

## Methods

After receiving institutional review board approval, a retrospective review of all consecutive NMS patients from a single tertiary institution who underwent PSF to the pelvis with bilateral SAI screw fixation from January 2010 to December 2021 was performed. Patients > 21 years old at the time of surgery, patients with greater or less than two SAI screws placed, or those with < 2 years of postoperative radiographic follow-up were excluded. Patient demographics, associated medical comorbidities, pre- and post-operative radiographic parameters, SAI screw size, and associated complications were recorded. All postoperative radiographs were evaluated to assess for evidence of SAI screw fracture, set screw dislodgement, or associated complications.

SAI screws were placed using the freehand technique. The appropriate start point was identified on the dorsal sacral ala, located just lateral to the midpoint of the S1 and S2 foramina. A curved awl was advanced toward the ipsilateral anterior inferior iliac spine, with a trajectory just superior to the sciatic notch on fluoroscopy. A teardrop view, obtained by rotating the fluoroscopy beam approximately 30° to the obturator oblique position, confirmed trajectory within the inner and outer tables. After crossing the sacroiliac joint, a flexible straight awl is used to develop a tract within the inner and outer tables, stopping adjacent to the anterior inferior iliac spine. A guidewire is placed into the established tract, the screw length is measured, and a cannulated screw is inserted. Cannulated poly-axial screws are used to ensure that the screw follows the appropriate tract and remains safely within the ilium, as in neuromuscular patients with poor bone quality, there is a greater likelihood for screw malposition if non-cannulated screws are used. Tapping was performed at the discretion of the surgeon. Screw diameter is selected by consideration of the patient’s height and weight, preoperative evaluation of advanced imaging when available (computed tomography (CT) or magnetic resonance imaging (MRI)), and intraoperative assessment of pelvic size on fluoroscopy. Safe screw trajectory is confirmed on anterior–posterior (AP) and obturator oblique views of the pelvis. Standard fixation at our institution involves the placement of bilateral S1 and L5 pedicle screws to achieve for 6 points of fixation at the spinopelvic junction.

Predictors of screw failure were investigated through Fisher’s exact test for categorical variables and Mann–Whitney rank sum test for continuous variables with an alpha level of 0.05. Statistical analysis was performed in Stata/IC (version 14.2, StataCorp LLC, College Station, TX).

## Results

One hundred forty-two patients met inclusion criteria. The average age at surgery was 13.6 (SD 2.7) years old and 40.8% of patients (58/142) were female. The predominant underlying diagnosis was cerebral palsy (85/142, 60%) (Table 1). The average pre-operative curve magnitude and pelvic obliquity were 84.4 (SD 29.1) degrees and 25.4 (SD 16.4) degrees, respectively. Of the 142 patients, ten (7.0%) were ambulatory. Average patient weight was 36.4 (SD 14.1) kilograms and body mass index (BMI) was 18.0 (SD 5.1) kg/m^2^ at the time of surgery.

**Table 1:** Demographics by underlying diagnosis

Diagnosis, *n* (%)	*N* = 142
Cerebral palsy	85 (60%)
Muscular dystrophy	29 (20%)
Spina bifida	4 (3%)
Spinal muscular atrophy	9 (6%)
Rett Syndrome	8 (6%)
Other	7 (5%)

**Fig. 1:**
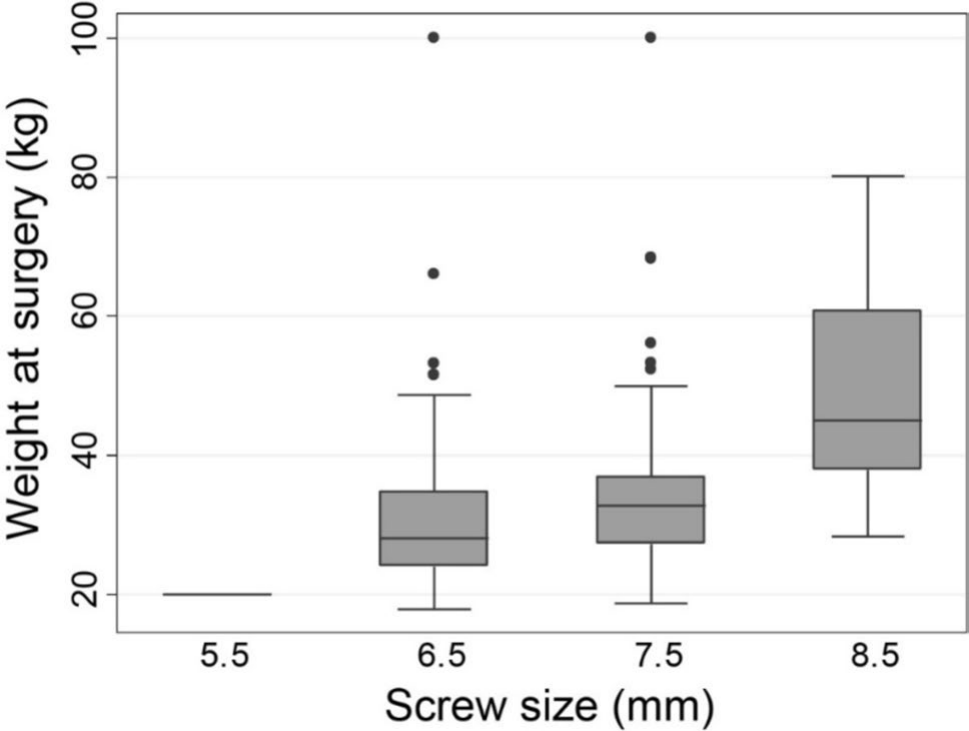
Trend towards larger SAI screw diameter with increasing patient weight

Posterior spinal fusion to the pelvis was performed with an average surgical time of 378 (SD 113) minutes and estimated blood loss of 826 (SD 488) milliliters. Postoperative curve magnitude measured 33 (SD 20) degrees, with an average correction of 51 (SD 22) degrees. Postoperative pelvic obliquity averaged 6 degrees (SD 7), with an average correction of 19 (SD 13) degrees. Bilateral SAI screws were placed in each patient, for a total of 284 screws (Table 2). Of the 284 screws, 55.6% (158/284) were 7.5mm screws. The average screw length was 72.7 (SD 10.6, range 50–100) mm. There was a trend towards increasing screw diameter with increasing patient weight (Fig. 1). Patient follow-up averaged 4.6 (SD 1.9) years after spinal fusion.

**Table 2:** SAI screw sizes and patient characteristics

Screw size	Count, *n* (%) *N* = 284	Age (years)	Weight (kg)	BMI (kg/m^2^)	Failures, *n*
5.5 mm	2 (0.7%)	10 ± 0	20.0 ± 0	12.4 ± 0	0
6.5 mm	74 (26.1%)	12.9 ± 2.5	32.0 ± 13.3	17.6 ± 4.9	0
7.5 mm	158 (55.6%)	13.7 ± 2.8	34.2 ± 11.2	17.3 ± 4.6	3 (1.9%): 2 set screws, 1 fracture
8.5 mm	50 (17.6%)	14.6 ± 2.3	50.3 ± 15.1	21.3 ± 5.9	2 (4.0%): 2 set screws

All values presented as mean ± standard deviation, unless otherwise noted

**Fig. 2:**
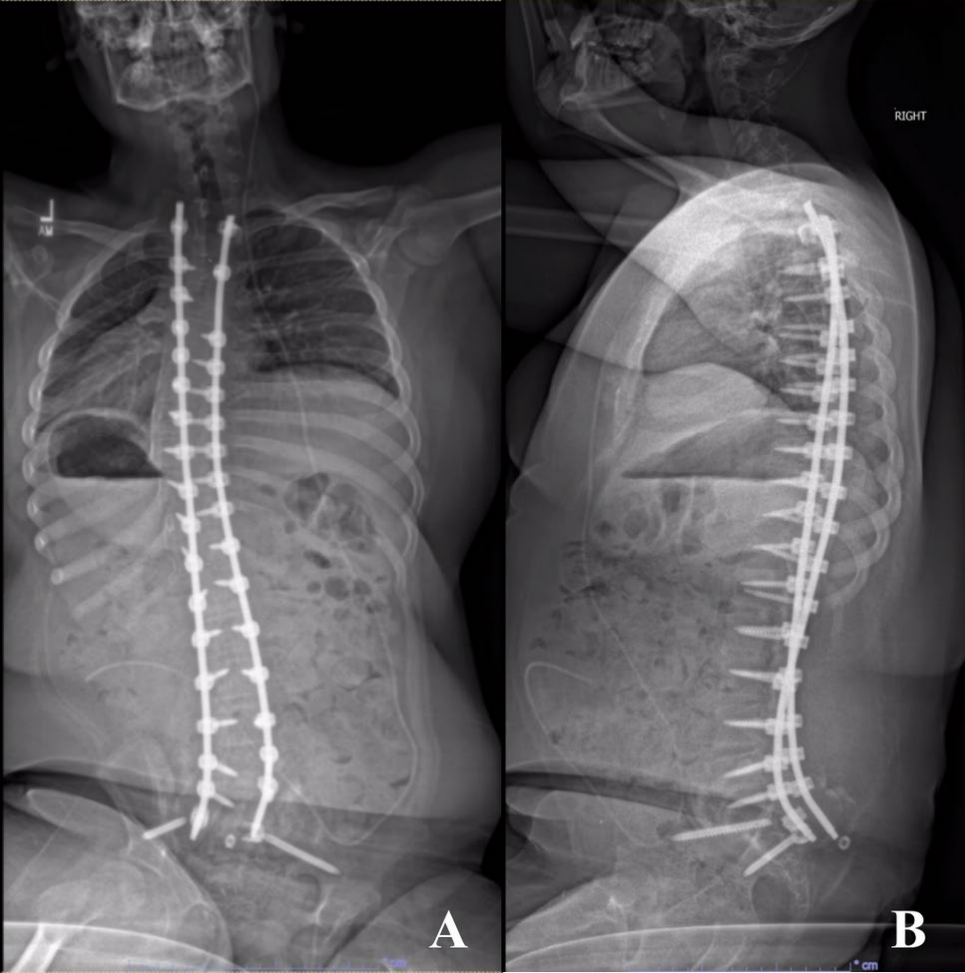
Posteroanterior (**A**) and lateral (**B**) sitting spine radiographs of a 15-year-old male with lumbar myelomeningocele treated with T2-Pelvis posterior spinal fusion. Follow-up radiographs at 1 year postoperatively demonstrated set screw dislodgement of the right SAI screw and fracture of the left SAI screw at the head-shaft junction. Both SAI screws were 7.5mm in diameter

Five screw failures (5/284, 1.8%), including four set screw dislodgements and one screw fracture, were identified in three (3/142, 2.1%) patients (Table 3). The average time from PSF to identification of failure was 8.7 (SD 5.1) months, and revision was uncommon, occurring in only one of these three patients. The first patient with screw failure, who had a lumbar myelomeningocele, developed a postoperative seroma that was treated with irrigation and debridement and paraspinal muscle flaps within two weeks of his index surgery. At the one-year postoperative visit he was noted to have right-sided set screw dislodgement and fracture of the left 7.5 mm SAI screw at the head-shaft junction (Fig. 2). He remained asymptomatic and had demonstrated stable alignment and thus was managed with continued observation. The second patient with SAI screw failure, who had paraplegia, had early dislodgement of bilateral SAI set screws identified at the 3-month postoperative visit and progressive sagittal imbalance for which he underwent surgical revision with replacement of the set screws. While there was no concern for infection at that time, he did develop a chronic infection within 6 months postoperatively and ultimately underwent implant removal after serial debridement. The remaining patient with a unilateral set screw dislodgement had fusion confirmed on a CT scan and was managed with continued observation. For the two patients managed with observation alone, they had a mean follow-up of 35 months after surgery and 24 months after fixation failure. During this time, the curve magnitude and pelvic obliquity remained essentially stable with a mean of – 1 degrees (range:  5 to + 4 degrees) change in curve magnitude and 3 degrees (range: + 1 to + 4 degrees) change in pelvic obliquity from the first postoperative films.

**Table 3:** SAI Screw Failure Case Summaries

Case	Etiology	Age (years), Sex	Weight (kg)	BMI (kg/m^2^)	SAI screw diameter (mm)	Failure type	Time to failure	Revision?
1	Spina Bifida	15, M	68.2	32.0	7.5	One set screw dislodged; One SAI screw fractured	13 months	No
2	C8 Paraplegia	14, M	56.3	28.7	8.5	Bilateral set screws dislodged	3 months	Yes. Set screws revised at 3 months. Implant removal for chronic infection at 4 years postop
3	Pelizaeus-Merzbacher Disease (Leukodystrophy)	15, M	34.9	15.0	7.5	One set screw dislodged	10 months	No

**Fig. 3:**
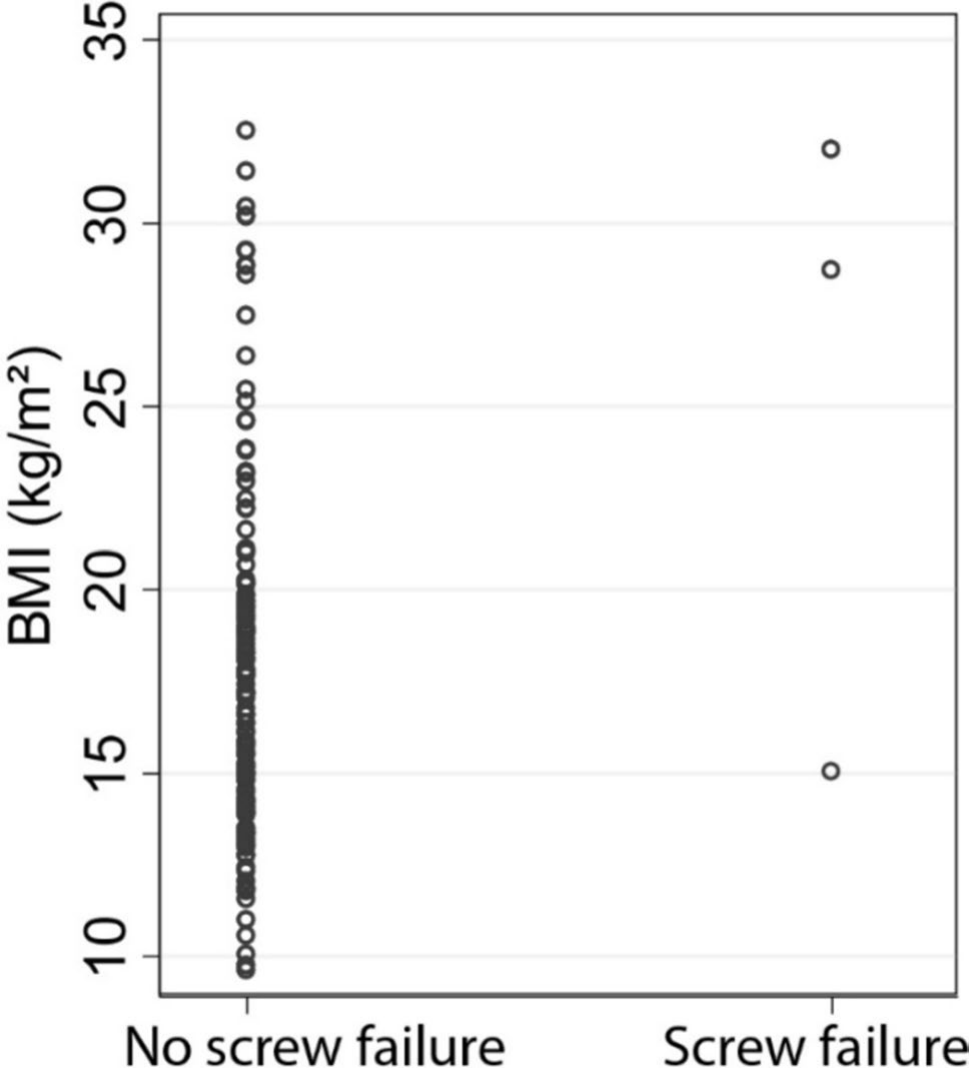
Patients with screw failure had higher BMI (* p* = 0.01)

Screw diameter was not significantly different for those that did and did not fail (*p* = 0.09). A higher BMI was associated with screw failure, with an average BMI of 27.3 versus 17.9 kg/m^2^ in those with and without screw failure (*p* = 0.01) (Fig. 3). Furthermore, ambulatory status was not predictive of failure (*p* = 1.0). While not reaching statistical significance, there was a trend towards longer surgical time (*p* = 0.06) and male gender (*p* = 0.08) in those who sustained screw failure.

## Discussion

This study describes the outcomes of SAI screw fixation in pediatric patients with neuromuscular scoliosis. The use of smaller SAI screws was common in this series, with all screws smaller than 9 mm diameter and 82.4% (234/284 screws) smaller than 8.5 mm diameter. Despite this, the overall rate of SAI screw failure was low, at 2.1%. Only one of these was a screw fracture while the majority (4/5) were from set screw dislodgement. It is unlikely that a larger screw diameter would have had an impact on the rate of set screw dislodgement. It is also notable that only one of three patients who developed SAI screw failure underwent revision.

As bending rigidity increases as a function of radius to the fourth power, maximizing screw diameter for pelvic fixation is a reasonable strategy to prevent screw fracture and somewhat intuitive. Early reports on the SAI technique recommended the use of screws > 9mm to help prevent fracture, both for adults and children, with recommendations based on small studies reporting early outcomes of the SAI screw technique [15, 16]. However, pediatric patients with neuromuscular scoliosis are often of small stature or develop atypical pelvic anatomy, both of which may narrow the size of the bony corridor between the inner and outer tables of the ilium. Thus, the use of such a large screw is often not feasible without risking an unsafe trajectory or fracture of the pelvis, and in our study less than 20.0% of screws were 8.5 mm or greater. Nevertheless, our screw fracture rate of < 1.0% is lower than previously reported rates of 3.1–11.2% in pediatric patients [11, 13, 14, 17]. In our practice, SAI screw size is chosen to maximize screw purchase while minimizing the risks of pelvic fracture. This is accomplished by review of available three-dimensional imaging preoperatively, intraoperative assessment of pelvic anatomy and consideration of the patient’s body weight. It is likely that this technique has allowed us to maximize screw length and diameter for the patient’s anatomy. If intraoperative navigation is available, this can also help optimize screw diameter, as demonstrated by Sullivan et al. [18].

As the use of these strategies to optimize screw diameter and length for patient anatomy are common, it is possible that the lower screw fracture rate in this study is reflective of the use of different implant systems. Differing screw morphology at the head/neck junction may predispose certain implant systems to fracture or set cap dislodgement due to different final tightening torques. In this study, two different implant systems were used. Due to the low rate of failure, system type was not assessed for significance. In addition, slight differences in surgical technique may be attribute to differences in failure rate. A more medial or lateral start point will change the angle between the head and shaft, and this may change the stresses on the screw neck, a concept also posited by Guler et al. [19]. While biomechanical analysis has found that head and shaft dissociation is the primary failure mode for poly-axial screws, the function of head/shaft angle on stresses at this junction is not fully understood [20].

Pediatric neuromuscular scoliosis patients are often smaller in size and have a lower functional demand. SAI screws of 8mm or greater diameter may not be necessary to support the neuromuscular patient’s physical demands. We found that patient age, weight, and BMI tended to increase as screw diameter increased. However, BMI was significantly greater in patients that experienced SAI screw failure and two of the patients that experienced SAI screw failure were either overweight or obese. In particular, the patient who developed a screw fracture had a body weight that was significantly greater than the average body weight of patients who received 7.5 mm SAI screws. It is possible that the additional body weight in this case was contributory to screw failure. In contrast, no patients with a similar body weight of 50kg with 8.5mm screws had a screw fracture identified. In addition to a careful evaluation of the patient’s pelvic anatomy, we recommend using the patient’s body weight to help guide screw selection: for patients with a body weight of 50kg or greater 8.5mm screws should be used if possible. Conversely, for patients with the body weight of 30kg or less, 6.5mm screws are likely robust enough to protect against failure.

Our standard spinopelvic fixation technique involves bilateral L5 and S1 pedicle screws and bilateral S2AI screws, achieving six total points of fixation about the spinopelvic junction and increasing rigidity at the distal end of the construct. Adult literature has shown similar findings, with additional rods or screws across the lumbosacral or sacroiliac junction improving rates of fixation failure [21, 22]. This strategy was not addressed in earlier reports on SAI screw failure, which may explain the lower rates of pelvic fixation failure in this series.

Set screw dislodgement was a more common implant-related complication, occurring in three patients (3/142, 2.1%). However, this rate remains quite low and similar to previously reported rates of 1.2–9.1% [9, 10, 14, 23]. Martin et al. reported a 5% rate of rod disengagement from SAI screws in adults and found that the magnitude of surgical correction was a risk factor for this [23]. In theory, a greater surgical correction increases the stress on the distal implants. Greater trunk mobility may also be contributory. While non-ambulatory status was not associated with failure in this series, two of the three who developed set screw dislodgement two could use their upper extremities for mobility. It is possible that the increased trunk motion of these patients created additional stress on the lower aspect of the implant construct and contributed to the failure. Particularly close attention should be paid to set screw tightening and maximizing screw length and diameter in these patients.

Revision following SAI screw failure remains uncommon. Only one of three patients underwent revision, following early bilateral set screw dislodgement with loss of correction. The remaining two patients who were asymptomatic demonstrated stable pelvic obliquity at 2 years after fixation failure. Revision rates of 0–33% are reported after SAI screw failure, supporting the notion that not all SAI screw failures are symptomatic or develop pseudarthrosis and require revision [9, 17]. As pseudarthrosis at the lumbosacral junction can occur, carefully assessing for this when SAI screw failure does present is important, as this may warrant surgical intervention. Most importantly, Mun et al. corroborated this with their 10-year follow-up study in which only 1/3 of patients with a screw fracture required revision. With durable correction of pelvic obliquity, they concluded that SAI screws are a favorable solution for pelvic fixation with a low complication rate that is maintained at 10 years [17].

To our knowledge, this is the largest study of pediatric neuromuscular scoliosis patients treated with bilateral SAI screw fixation. However, given the infrequent nature of screw fracture in our patient cohort, we were unable to calculate a risk factor analysis related to screw failure, such as rod type, GMFCS level (where applicable), or curve characteristics. Nevertheless, our low failure rate does help prove the concept that smaller-diameter screw sizes can be used successfully. While our study does incorporate a large population of pediatric neuromuscular scoliosis patients of varying etiologies, ages, BMI, and curve severity, the use of a single site limits our sample size and may also impact the generalizability of our results to other institutions’ patient populations. Finally, our length of follow-up would not have captured long-term implant-related complications, though previous reports have shown that mid-term complication rates are stable at long-term follow-up [17]. Our mean time to implant failure was less than 9 months which further supports that theory.

In conclusion, SAI screw fracture is rare after PSF to pelvis in pediatric patients with neuromuscular scoliosis. Contrary to pelvic fixation in adults, the use of smaller diameter SAI screws, which is often advantageous in patients with smaller anatomy, was not associated with increased risk of screw failure. We recommend careful preoperative and intraoperative evaluation of the patient’s pelvic anatomy as the variable sizes of these patients benefit from a more tailored approach.

## Data Availability

Raw data were generated at Children's Hospital Los Angeles. Derived data supporting the findings of this study are available from the corresponding author (LA) on request.
